# HbA_1c_ screening for new onset diabetes following acute coronary syndrome: is it a worthwhile test in clinical practice?

**DOI:** 10.1186/s40200-017-0296-4

**Published:** 2017-04-04

**Authors:** Sebastian T. Lugg, Christine J. H. May, Peter Nightingale, Robbie P. E. Tuffley, June Al-Hourani, Parijat De

**Affiliations:** 1 Department of Diabetes & Endocrinology, City Hospital Birmingham, Sandwell & West Birmingham NHS Trust, Birmingham, UK; 2 Institute of Inflammation and Ageing, Center for Translational Inflammation Research, University of Birmingham, Birmingham, UK; 3 Institute of Translational Medicine, University Hospitals Birmingham NHS Foundation Trust, Birmingham, UK

**Keywords:** Type 2 diabetes, Pre-diabetes, HbA_1c_, Acute coronary syndrome

## Abstract

**Background:**

Diabetes and pre-diabetes are prevalent in acute coronary syndrome (ACS) and relate to adverse outcomes. This study used HbA_1c_ to screen for degrees of glucose intolerance amongst patients without known diabetes presenting with ACS.

**Methods:**

Over a 1-year period (June 2014–2015) consecutive patients admitted to a single centre cardiology unit with an initial diagnosis of ACS without prior diabetes diagnosis were electronically referred to our diabetes team. Patients were screened for the presence of diabetes by use of an initial HbA_1c_ blood test on day 2 or 3 post admission. If abnormal (≥42 mmol/mol; ≥6.0%), patients were invited for a repeat HbA_1c_ at 2 weeks, and if an intermediate result (42–47 mmol/mol; 6.0–6.4%), for an oral glucose tolerance test (OGTT) at 3 months. Patients were diagnosed with Type 2 diabetes if the repeat HbA_1c_ result was high (≥48 mmol/mol; ≥6.5%) or the OGTT at 3 months confirmed the diagnosis. Other data collected included baseline demographics, risk factors and any history of cardiovascular disease. All patients with ACS were stratified according to the diagnosis and subsequent management.

**Results:**

We screened 399 patients in total. The mean age was 65 ± 14 years, 268 (67%) were men, 290 (73%) were Caucasian, 95 (24%) were South Asian and 14 (4%) were Afro-Caribbean ethnicity. Of all patients, 57 (14.3%) were diagnosed as pre-diabetes and 43 (10.8%) newly diagnosed diabetes. During the study 28 (7%) patients could not be classified; 6 (1.5%) patients died during the study and 22 (5.5%) patients were missing either initial or repeat HbA_1c_ and were subsequently lost to follow up. Of the baseline variables assessed, there were significantly more patients of South Asian ethnicity in the diabetes group compared to the normal group (42 vs 20%; *p* = 0.003). There was no difference in detection rates in patients with more severe ACS requiring percutaneous or cardiac surgical intervention.

**Conclusions:**

The use of a simple HbA_1c_ screening method in clinical practice can detect new onset diabetes in approximately 1 in 10 high-risk post ACS patients.

## Background

It is estimated that more than 1 in 16 people in the UK have diabetes mellitus; diagnosed or undiagnosed. Type 2 diabetes is recognised as a major cardiovascular risk factor and its close relationship with cardiovascular morbidity and mortality is well established [1]. Indeed, cardiovascular disease has been accounted for 52% of deaths in Type 2 diabetes [2]. Irrespective of the presence or absence of diagnosed diabetes, disturbances of glucose metabolism are widely prevalent in patients presenting with acute coronary syndrome (ACS). Even in those patients without established Type 2 diabetes, ACS patients with glucometabolic dysregulation have an increased risk of mortality both in hospital and after discharge [3–6].

Screening ACS patients on admission would enable both early detection and management of glucose intolerance and potentially improve patient outcomes. Most recent guidelines by the European Society of Cardiology in collaboration with European Association for the Study of Diabetes have recommended that all ACS patients are screened for Type 2 diabetes [7]. Current screening methods using fasting plasma glucose (FPG) or the oral glucose tolerance test (OGTT) have limitations in the acute setting. In 2012 the World Health Organisation (WHO) approved the use of HbA_1c_ as the preferred screening test in the diagnosis and targeted screening for Type 2 diabetes [8]. The National Institute of Health and Care Excellence (NICE) recommend that all ACS patients with admission blood glucose concentrations above 11.0 mmol/l should have FPG no earlier than day 4 after ACS onset or have an HbA_1c_ test before discharge [9]. A screening strategy using HbA_1c_ as the preferred test would be pragmatic and improve early detection and management of glucose intolerance in acute cardiology care practice [10–12].

The aim of our study was to audit the effectiveness of a simple screening programme based on HbA_1c_ levels alone to identify degrees of glucose intolerance amongst non-diabetes patients presenting with ACS.

## Methods

This prospective audit was conducted at a single-centre over a 1-year period (June 2014–2015). Consecutive patients admitted to a cardiology unit with an initial diagnosis of ACS without prior diabetes diagnosis were included. All patients had a random HbA_1c_ on day 2 or 3 following admission and were simultaneously referred to the diabetes team via Think Glucose, an electronic referral system using iCM software, which was already in place within our Trust. Our study to audit the effectiveness of the established screening tool in clinical practice was registered with Sandwell and West Birmingham NHS Trust audit department (audit code 674).

**Fig. 1:**
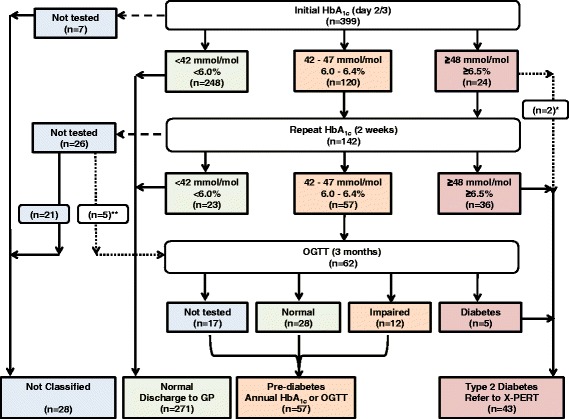
Study protocol including patient numbers for initial and repeat HbA_1c_ test, and then oral glucose tolerance test (OGTT). *2 patients were diagnosed with Type 2 diabetes as initial HbA_1c_ levels ≥ 99 mmol/mol with presence of symptoms; **5 patients attended OGTT at 3 months despite missing their repeat HbA_1c_ at 2 weeks

Patients were managed according to a protocol (Fig. 1). HbA_1c_ was measured in our hospital laboratory by the Tosoh GA analyser using HPLC method (normal range between 28 and 42 mmol/mol). Initial HbA_1c_ were categorised as normal (<42 mmol/mol; <6.0%), intermediate range (between 42 and 47 mmol/mol; 6.0% and 6.4%) or high (≥48 mmol/mol; ≥6.5%) according to our NICE screening guidance [9]. Patients with an intermediate or high initial HbA_1c_ were invited for a repeat HbA_1c_ at 2 weeks as per American Diabetes Association guidelines to confirm diagnosis of Type 2 diabetes for those with high levels [13]. Patients with a repeat HbA_1c_ within intermediate range were further categorised by an OGTT at 3 months as previously recommended [10]. This was a standardised 75-g OGTT, performed in the morning, after a 12-h overnight fast, and results were defined as normal, impaired or consistent with diabetes diagnosis, according to the WHO 1998 definitions [14]. Any abnormal test results were acted upon.

Data collected included baseline demographics of age, sex, ethnicity and smoking status. Risk factors for cardiovascular disease were also collected including background of hypercholesterolaemia, hypertension, peripheral vascular disease, cerebrovascular disease, myocardial infarction, and any previous percutaneous coronary intervention (PCI) or coronary artery bypass graft (CABG). Other outcome data collected included high sensitivity cardiac troponin T, which was measured on admission and at least 12 h after the primary clinical event and admission creatinine level. The final ACS diagnosis on hospital discharge was recorded as ST-elevation myocardial infarction (STEMI), non-ST elevation MI (NSTEMI), unstable angina, or not ACS if investigations excluded the initial diagnosis. Subsequent management was recorded and included medical therapy, percutaneous coronary intervention, and referral for urgent coronary artery bypass graft. Inpatient mortality was recorded, as were the data of those patients who did not return for follow up testing.

### Statistical analysis

Results are expressed as mean (SD) for continuous variables and as a percentage for categorical variables. A paired *t*-test was used to compare initial and repeat HbA_1c_. The baseline characteristics and outcomes of patients diagnosed as normal, pre-diabetes and diabetes were compared using Kruskal-Wallis (KW) for continuous variables and Fisher’s exact test (FET) for categorical variables. Where the comparison of the three groups was significant (*p* < 0.05), pairwise comparisons were performed. Pairwise comparisons for KW were adjusted for multiple comparisons; those for FET were not, therefore pairwise comparisons for FET were treated as significant only if *p* < 0.0167. Statistical analysis was performed using SPSS Statistics for Windows, Version 22.0. Armonk, NY: IBM Corp.

## Results

### Initial HbA_1c_

Of the 399 patients in our study, results of initial HbA_1c_ were normal in 248 (62.2%) patients. Results were intermediate in 120 (30.1%) patients and high in 24 (6%) patients. Of those patients with an initial high HbA_1c_, 2 patients had HbA_1c_ levels of 99 and 127 and were subsequently diagnosed with Type 2 diabetes due to the markedly elevated level and the presence of symptoms. There were 7 (1.8%) patients that did not have an initial HbA_1c_; 1 (0.3%) patient died following referral and 6 (1.5%) patients were initially referred by the cardiology team without HbA_1c_ measurements and were subsequently lost to follow up.

### Repeat HbA_1c_

**Fig. 2:**
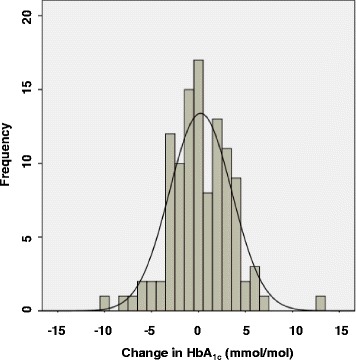
Histogram demonstrating the difference in HbA_1c_ levels between initial and repeat test (Repeat minus the initial HbA_1c_ level) in those initially identified as having HbA_1c_ ≥ 42 mmol/mol

Of the 142 patients due for repeat HbA_1c_ at 2 weeks, results of the repeat HbA_1c_ were normal in 23 (16.2%) patients, intermediate in 57 (40.1%) patients, and diagnosed Type 2 diabetes in 36 (25.4%) patients. There were 26 (18.3%) patients that did not have a repeat test of which; 5 (3.5%) patients died and 21 (14.8%) patients did not attend the follow up HbA_1c_. The difference in HbA_1c_ level between the initial and repeat test was normally distributed (Fig. 2), with a mean (±SD) difference of 0.23 (±3.31) mmol/mol. There were no significant differences between the initial HbA_1c_ and the repeat HbA_1c_ levels (*p* = 0.506).

### OGTT at 3 months

There were 62 patients scheduled to attend an OGTT at 3 months, this included 57 patients with a repeat intermediate HbA_1c_ and 5 patients who attended despite missing their repeat HbA_1c_ test. OGTT result was normal in 28 (45.2%) patients, impaired in 12 (19.4%) patients, and diagnosed diabetes in 5 (8.1%) patients. A total of 17 (27.4%) patients did not attend the OGTT. Following OGTT, patients with an impaired or normal result, or those who were not tested were all still classified as pre-diabetes due to the prior intermediate repeat HbA_1c_ result. Therefore a letter was sent to their GP advising the patient to have annual HbA_1c_ levels or OGTT checked.

### Final outcome

The end result of screening process diagnoses pre-diabetes in 57 (14.3%) patients and Type 2 diabetes in 43 (10.8%) patients. During the study 28 (7%) patients could not be classified; 6 (1.5%) patients died during the study and 22 (5.5%) patients were missing either initial or repeat HbA_1c_ and were subsequently lost to follow up.

### Baseline characteristics and outcome

Of all the baseline characteristics studied in our prospective study, the only characteristics that were significantly different were ethnicity and sex (Table 1). On univariate analysis, there appeared to be significantly fewer men in the pre-diabetes group; however pairwise comparisons between groups did not reach the level of significance required (*p* < 0.0167). There were significantly more patients who were South Asian in the Type 2 diabetes group compared to the normal group (42 vs 20%; *p* = 0.003). Significantly fewer patients who were Caucasian had diabetes compared to the normal group (53 vs 78%; *p* = 0.001). Interestingly there was no difference in the severity of ACS or management required between patients with normal results, or those who were diagnosed as pre-diabetes or Type 2 diabetes (Table 2).

**Table 1:** Baseline characteristics of all patients and those in normal, pre-diabetes and Type 2 diabetes groups

	All patients	Normal	Pre-diabetes	Type 2 Diabetes	Not classified	*P*-value
	(*n* = 399)	(*n* = 271)	(*n* = 57)	(*n* = 43)	(*n* = 28)	
Initial HbA_1c_ (mmol/mol)		39 (36–40) ^b,c^	44 (43–46) ^a^	47 (46–50) ^a^	44 (43–47)	<0.001 KW
Repeat HbA_1c_ (mmol/mol)		40 (38–41) ^b,c^	44 (43–46) ^a,c^	50 (48–51) ^a,b^		<0.001 KW
Age (years)	65 (61–79)	64 (54–76)	70 (60–76)	64 (55–74)	76 (47–82)	0.276 KW
Sex (% Men)	268 (67%)	187 (69%)	31 (54%)	33 (77%)	17/28 (61%)	0.043 FET
Ethnicity:						
Caucasian (%)	290 (73%)	212 (78%) ^c^	36 (63%)	23 (53%) ^a^	19 (68%)	0.002 FET
South Asian (%)	95 (24%)	53 (20%) ^c^	17 (30%)	18 (42%) ^a^	7 (25%)	
Afro-Caribbean (%)	14 (4%)	6 (2%)	4 (7%)	2 (5%)	2 (7%)	
Current Smoker (%)	84/386 (22%)	62/260 (24%)	10/56 (18%)	7/42 (17%)	5/28 (18%)	0.481 FET
Hypercholesterolaemia (%)	162/383 (42%)	103/257 (40%)	28/55 (51%)	20/43 (47%)	11/28 (39%)	0.295 FET
Cholesterol level (mmol/l)	4.6 (3.4–5.6)	4.4 (3.7–5.3)	4.7 (3.6–5.2)	4.4 (3.4–5.5)	3.9 (3.1–5.3)	0.974 KW
Hypertension (%)	200/387 (52%)	124/260 (48%)	34/56 (61%)	25/43 (58%)	17/28 (61%)	0.123 FET
PVD (%)	22/399 (6%)	12/267 (4%)	3/57 (5%)	3/43 (7%)	3/28 (11%)	0.674 FET
Previous stroke/TIA (%)	29/399 (7%)	19 (7%)	3 (5%)	4 (9%)	3 (11%)	0.673 FET
Cardiovascular disease:						
Myocardial infarction (%)	94/396 (24%)	63/271 (23%)	13/56 (23%)	11/43 (26%)	7/27 (26%)	0.920 FET
Previous PCI (%)	72/399 (18%)	47 (17%)	13 (23%)	6 (14%)	6 (21%)	0.479 FET
Previous CABG (%)	26/399 (7%)	17 (6%)	5 (9%)	2 (5%)	2 (7%)	0.779 FET

*KW* kruskal-wallis, *FET* fisher’s exact test, *TIA* transient ischaemic attack, *PVD* peripheral vascular disease, *CABG* coronary artery bypass graft, *PCI* percutaneous coronary intervention. The not classified group were excluded from the analysis. Where the comparison of the three groups is significant (*p* < 0.05), pairwise comparisons were performed. Pairwise comparisons for KW are adjusted for multiple comparisons, those for FET were not, therefore pairwise comparisons for FET were treated as significant only if *p* < 0.0167. Superscript letters (^a-c^) were used; (^a^) to mean significantly different from Normal group, (^b^) to mean significantly different from Pre-diabetes group and (^c^) to mean significantly different from Type 2 diabetes group

**Table 2:** Cardiovascular outcome in normal, pre-diabetes and Type 2 diabetes groups

		Normal	Pre-diabetes	Type 2 Diabetes	Not classified	*P*-value
		(*n* = 271)	(*n* = 57)	(*n* = 43)	(*n* = 28)	
Creatinine (micromol/l)		82 (70–101)	83 (71–97)	80 (68–101)	99 (79–136)	0.865 KW
Troponin T (ng/l)	Initial	52 (20–242)	52 (22–138)	67 (15–244)	60 (35–1980)	0.892 KW
	Repeat	158 (37–721)	83 (36–514)	113 (29–1167)	79 (33–3050)	0.551 KW
	% Change	44 (1–312)	19 (0–273)	46 (1–238)	21 ((−2) − 145)	0.541 KW
ACS diagnosis	STEMI	100 (37%)	15 (26%)	11 (26%)	9 (32%)	0.485 FET
	NSTEMI	100 (37%)	25 (44%)	21 (49%)	9 (32%)	
	Unstable angina	29 (11%)	8 (14%)	6 (14%)	3 (11%)	
	Not ACS	42 (15%)	9 (16%)	5 (12%)	7 (25%)	
Management	Nil	39 (14%)	9 (16%)	5 (12%)	7 (25%)	0.753 FET
	Medical	57 (21%)	16 (28%)	7 (16%)	5 (18%)	
	PCI	149 (55%)	26 (46%)	27 (63%)	14 (50%)	
	CABG	26 (10%)	6 (11%)	4 (9%)	2 (7%)	

*KW* kruskal-wallis, *FET* fisher’s exact test, *ACS* acute coronary syndrome, *STEMI* ST elevation myocardial infarction, *NSTEMI* non-ST elevation myocardial infarction, *PCI* percutaneous coronary intervention, *CABG* coronary artery bypass graft

## Discussion

Our screening programme based solely on HbA_1c_ blood tests to detect diabetes and pre-diabetes in high-risk ACS patients found approximately 1 in 10 patients with incident diabetes. This concurs with the study by Arnold et al. [15], that found among 2854 acute myocardial infarction patients without known diabetes on admission, there were 287 patients (10%) that met criteria for Type 2 diabetes (defined by a core laboratory glycated haemoglobin of ≥6.5%). They found that 2 of 3 patients with newly diagnosed diabetes were unrecognized by treating clinicians, receiving neither diabetes education, glucose-lowering medications at discharge, nor documentation of diabetes in the chart. Conversely, in our cohort, all the newly diagnosed patients received education and were seen in specialist clinic with regards to starting glucose lowering medication.

In patients with established coronary artery disease, a large study of 4004 patients has compared the screening capacity of FPG, 2-h post load post plasma glucose (2hPG), OGTT (FPG & 2hPG) and HbA_1c_ [16]. In this study 29% had undetected diabetes by the use of all screening tests. Out of them, the proportion identified by FPG was 75%, by 2hPG 40%, by HbA_1c_ 17%, by FPG + HbA1c 81%, and by OGTT FPG+ 2hPG 96%. Interestingly, only 7% of diabetes were detected by all three methods of FPG, 2hPG, and HbA_1c_. In patients with ACS, previous studies investigating the prevalence of undiagnosed Type 2 diabetes using OGTT during admission (or following discharge) have found higher absolute proportions compared to our study (20–30%) [5, 17, 18]. The higher percentage of diagnosis is likely due to the OGTT having previously shown to have higher a sensitivity for detecting Type 2 diabetes than HbA_1c_ in patients with ACS [19, 20]. Therefore, the results from our study in relying on HbA_1c_ alone, may be missing around 8% of patients with undetected diabetes. However, the alternative strategies of detecting diabetes in ACS patients have their limitations in clinical practice. Firstly, the FPG can be acutely elevated and therefore can be unreliable in the first 2 days after a myocardial infarction [20]. Secondly, the OGTT test can also be affected by multiple factors, which include carbohydrate diet and physical activity levels prior to the test as well as the severity of myocardial damage and timing of the test in relation to an index event [20]. The main limitation of the OGTT however, as found in our pilot study, is that it is resource intensive. The Euro Heart Survey found that the recommended OGTT was performed only in 56% of the patients [21]; this could be explained by the ethical permits to perform an OGTT not being issued in some countries, technical obstacles experienced in the cardiology care setting for these not-as-routine measures, and finally overt fasting hyperglycaemia that was considered sufficient to establish the diagnosis of diabetes.

The use of initial HbA_1c_ test has several advantages over FPG or an OGTT in the acute setting. Predominantly, as the test can be performed in the non-fasting state and reflects average glucose concentration over the preceding 2–3 months and is therefore not affected by stress-induced changes in blood glucose levels. Indeed, we have demonstrated that repeat levels within 2 weeks do not show significant changes. HbA_1c_ has also been shown to independently predict glucose intolerance at 3 months in patients admitted with ACS without known diabetes [odds ratio (95% CI): 2.58 (1.17–6.09) *p* = 0.024], and correlates with 2hPG and OGTT [16]. We repeated the HbA_1c_ at 2 weeks, although this has been previously recommended at 4–8 weeks mainly on logistical grounds, fully recognising that a one-off test would have perhaps been sufficient for diagnostic purposes [10]. We found that repeat testing at 2 weeks was feasible, practical and a reassuring test to confirm the diagnosis and promptly inform patients of the diagnosis of potential glucose abnormalities.

Given the association with elevated HbA_1c_ and mortality, Gholap et al. recommend performing an OGTT at 4–8 weeks post-discharge in those with HbA_1c_ between 42 and 47 mmol⁄mol (6.0 and 6.4%) for accurate categorisation of glucose intolerance [10]. Performing OGGT in patients with HbA_1c_ <42 mmol/mol (<6.0%) was not recommended, and this is supported by NICE guidelines [9]. Gholap et al. made recommendations that these patients should be followed up with annual HbA_1c_ measurements; however there is limited evidence available for this at present. We felt that indefinite annual HbA_1c_ measurements of these individuals may strain services, and wanted to emphasise annual follow up in those patients who had abnormal HbA_1c_ and OGTT not meeting criteria for diabetes diagnosis.

Other than known diabetes, there were no other exclusion criteria to the patients involved in our study. There are limitations to the HbA_1c_ test, which includes the influence of red cell survival; any condition that shortens erythrocyte survival or decreases mean erythrocyte age may falsely lower HbA_1c_ test results regardless of the assay method used [22]. Furthermore, we did not exclude patients on the basis of chronic kidney disease severity; however some studies have shown that although the HbA_1c_ test performs well in milder chronic kidney disease, the accuracy of the test in patients with severe nephropathy requires further investigation [23].

We found a significantly high proportion of dysglycaemia in the South Asian population. This finding is in keeping with other studies demonstrating that Asian Indians without prior diagnosed diabetes show a high prevalence of hyperglycaemia following ACS [24]. An important factor, which may be contributing to this finding, is that HbA_1c_ concentrations have been shown to be higher in some ethnic groups (Afro-Caribbean, Hispanic, Asian) compared to Caucasian patients with similar plasma glucose levels [25]. Our study also found that the incidence of pre-diabetes and Type 2 diabetes did not differ between patients with differing severity of ACS. This is supported by previous studies that have shown patients with stable and unstable coronary artery disease [20], as well as patients with cerebro- and peripheral vascular disease have about the same proportion of previously unrecognised diabetes [26]. This further supports that this screening programme should be utilised for all suspected ACS patients, regardless of severity. Snir et al., found that in a multicentre observational study of 1743 patients, only 41% of diabetes patients admitted with ACS have HbA_1c_ measured in hospital [27]. There was a great variation in practice in different centres from 7.7 to 87.6%; those patients who were tested were more likely to have STEMI and referred for cardiac catheterisation.

### Limitations

This is a real life prospective study utilising a simple screening programme in ACS patients without prior diabetes diagnosis, involving patients of all ages and associated comorbidities seen in day-to-day clinical practice. We accept that including more comorbid patients may have increased the prevalence of glucometabolic disturbances in our study, though we did not find any significant differences in pre-diabetes and diabetes in patients with other cardiovascular comorbidities including hypertension, cerebro- and peripheral vascular disease. Data is incomplete for some patients, as they were lost to follow up. However, only a minority of patients (5.5%) did not receive an outcome. Our study showed a larger number of patients did not attend their OGTT; approximately 1 in 3 patients did not attend follow up appointment at 3 months. These patients were already recognised as high risk following their initial HbA_1c_ result and were recommended annual follow up for this reason. Furthermore, we acknowledge that not all patients presenting with ACS within the time period would have been tested and referred, as we only captured data of those patients who were referred via our Think Glucose iCM referral system. Our cardiology ward admits approximately 600 patients per year with suspected ACS; therefore we have been successful in screening 2 in 3 patients admitted, with some of the remaining patients not be suitable for screening because of pre-existing diabetes diagnosis. Finally, we have used HbA_1c_ as a screening tool in a relatively acute post ACS situation where its validity has not been robustly tested but we feel that the practical difficulties and limitations FPG and OGTT offers, HbA_1c_ is still a worthwhile, simple and practical screening option.

## Conclusions

We have screened our post ACS patients with a simple and practical random HbA_1c_ blood test. This method has detected approximately 1 in 7 patients with pre-diabetes and 1 in 10 with diabetes in high-risk post ACS patients, who would otherwise have been missed, with potential implications. Patients of South Asian ethnicity presenting with ACS are at highest risk of developing Type 2 diabetes. All this fits in with the global drive towards screening and detecting new onset Type 2 diabetes in high-risk populations. However, further prospective studies are needed to address the role of HbA_1c_ in strategies to accurately screen for every new patient with diabetes post ACS.

## Abbreviations

2hPG: 2-h post load post plasma glucose
ACS: Acute coronary syndrome
CABG: Coronary artery bypass graft
FET: Fisher’s exact test
FPG: Fasting plasma glucose
KW: Kruskal-Wallis
NICE: National institute of health and care excellence
NSTEMI: Non-ST elevation myocardial infarction
OGTT: Oral glucose tolerance test
PCI: Percutaneous coronary intervention
STEMI: ST elevation myocardial infarction
WHO: World health organization
